# Plasma MicroRNA Signature Validation for Early Detection of Colorectal Cancer

**DOI:** 10.14309/ctg.0000000000000003

**Published:** 2019-01-25

**Authors:** Marta Herreros-Villanueva, Saray Duran-Sanchon, Ana Carmen Martín, Rosa Pérez-Palacios, Elena Vila-Navarro, María Marcuello, Mireia Diaz-Centeno, Joaquín Cubiella, Maria Soledad Diez, Luis Bujanda, Angel Lanas, Rodrigo Jover, Vicent Hernández, Enrique Quintero, Juan José Lozano, Marta García-Cougil, Ibon Martínez-Arranz, Antoni Castells, Meritxell Gironella, Rocio Arroyo

**Affiliations:** 1 Advanced Marker Discovery (Amadix), Valladolid, Spain;; 2 Gastrointestinal & Pancreatic Oncology Group, Centro de Investigación Biomédica en Red de Enfermedades Hepáticas y Digestivas (CIBERehd)/Hospital Clinic de Barcelona/Institut d'Investigacions Biomediques August Pi I Sunyer (IDIBAPS), Barcelona, Spain;; 3 Department of Gastroenterology, Complexo Hospitalario Universitario de Ourense, Instituto de Investigación Sanitaria Galicia Sur, CIBERehd, Ourense, Spain;; 4 Department of Gastroenterology, Hospital Universitario de Burgos, Burgos, Spain;; 5 Department of Gastroenterology, Hospital Donostia/Instituto Biodonostia (CIBERehd). Universidad del País Vasco UPV/EHU, San Sebastián, Spain;; 6 Department of Gastroenterology, Hospital Clínico Universitario, IIS Aragón, University of Zaragoza, CIBERehd, Zaragoza, Spain;; 7 Gastroenterology Unit, Hospital General Universitario de Alicante, Alicante, Spain;; 8 Department of Gastroenterology, Complexo Hospitalario Universitario de Vigo, Vigo, Spain;; 9 Department of Gastroenterology, Hospital Universitario de Canarias, Universidad de La Laguna, Instituto Universitario de Tecnologías Biomédicas (ITB) and Centro de Investigación Biomédica de Canarias (CIBICAN), San Cristobal de La Laguna, Tenerife, Spain;; 10 Bioinformatics Platform, CIBERehd, Barcelona, Spain;; 11 OWL Metabolomics, Parque Tecnológico de Bizkaia, Derio, Bizkaia, Spain.

## Abstract

Specific microRNA (miRNA) signatures in biological fluids can facilitate earlier detection of the tumors being then minimally invasive diagnostic biomarkers. Circulating miRNAs have also emerged as promising diagnostic biomarkers for colorectal cancer (CRC) screening. In this study, we investigated the performance of a specific signature of miRNA in plasma samples to design a robust predictive model that can distinguish healthy individuals from those with CRC or advanced adenomas (AA) diseases.

**METHODS:**

Case control study of 297 patients from 8 Spanish centers including 100 healthy individuals, 101 diagnosed with AA, and 96 CRC cases. Quantitative real-time reverse transcription was used to quantify a signature of miRNA (miRNA19a, miRNA19b, miRNA15b, miRNA29a, miRNA335, and miRNA18a) in plasma samples. Binary classifiers (Support Vector Machine [SVM] linear, SVM radial, and SVM polynomial) were built for the best predictive model.

**RESULTS:**

Area under receiving operating characteristic curve of 0.92 (95% confidence interval 0.871–0.962) was obtained retrieving a model with a sensitivity of 0.85 and specificity of 0.90, positive predictive value of 0.94, and negative predictive value of 0.76 when advanced neoplasms (CRC and AA) were compared with healthy individuals.

**CONCLUSIONS:**

We identified and validated a signature of 6 miRNAs (miRNA19a, miRNA19b, miRNA15b, miRNA29a, miRNA335, and miRNA18a) as predictors that can differentiate significantly patients with CRC and AA from those who are healthy. However, large-scale validation studies in asymptomatic screening participants should be conducted.

## INTRODUCTION

Colorectal cancer (CRC) is the third most commonly diagnosed cancer and the fourth leading cause of cancer-related death worldwide and is expected to increase by 60% by 2030 (1).

Although some strategies are available to screen average risk patients including fecal occult blood testing (FOBT) (immunochemical test) alone or combined with stool DNA examination, endoscopy (sigmoidoscopy or colonoscopy), and a blood-based test to evaluate biomarkers, each one of them have important disadvantages (2). Current fecal test has the advantages of cut-off that can be adjusted and low price whereas stool DNA test has shown to detect significantly more cancers than fecal test and better sensitivity for advanced adenomas (AA). By contrast, lower specificity demands more colonoscopy resources being invasive and costly. Sigmoidoscopy and colonoscopy offer direct visualization and detection of a colonic polyps or advanced neoplasia with the advantage of getting a pathology specimen.

Only adequate biomarkers used in a screening setting will allow detecting precancerous adenomas (called AA) that could be removed during colonoscopy, reducing cancer incidence or also detecting malignant lesions at stages where cure is possible. To achieve higher levels of adherence to CRC screening, accurate blood-based test seems to be the best strategy (3).

MicroRNAs (miRNAs) are 18–22 nucleotide noncoding RNAs that post-transcriptionally regulate gene expression and control various cellular mechanisms including tumorigenesis and the development of various types of cancers (4–6). Accumulating evidence supports the existence of specific miRNA in biological fluids that can facilitate earlier detection of the tumors becoming then diagnostic biomarkers. In particular, circulating miRNAs have also emerged as promising diagnostic biomarkers for CRC (5). For instance, several authors demonstrated that miR21 in serum is a promising biomarker for the early detection and prognosis of CRC (7,8). More recently, miR-200c and miR-203 implicated in epithelial-to-mesenchymal transition have been described as noninvasive biomarker for CRC prognosis and to predict metastasis (9,10). Fecal miR-106a was shown to be a useful maker for patients with CRC negative for immunochemical fecal test (11). Because some other publications reported different accuracy values when various miRNAs are used as biomarkers, reproducible and confirmatory studies are required to validate analytical and clinical robustness.

In line with these innovative data, we demonstrated in a previous study (12) that patients with CRC and AA have significantly different patterns of miRNA expression than healthy individuals. A set of miRNAs (i.e., miRNA19a, miRNA19b, miRNA15b, miRNA29a, miRNA335, and miRNA18a) is upregulated, having discriminative capacity when found in plasma samples.

We hypothesized that a specific signature of miRNA could be used for CRC screening purposes.

Based on our previous work, our aim here was to design and validate a robust predictive model that can distinguish healthy individuals from those with advanced neoplasm (i.e., CRC or AA) in a larger cohort to confirm clinical validity.

## METHODS

### Study subjects

A total of 300 Caucasian subjects from 8 Spanish hospitals were enrolled for a case–control study (AMD-MCR-2014-01) conducted from May 2014 to June 2016. The study was approved by respective Clinical Research Ethics Committee. All participants provided written informed consent. The study was designed to develop and validate a new test for CRC screening after a biomarker discovery phase conducted previously in a series of 196 patients (12).

Inclusion criteria included controls, AA and a reduced number of CRC subjects from a CRC screening program referred to colonoscopy after a fecal immunochemical test (FIT) positive result, and additional clinically diagnosed CRC scheduled for surgery (majority of CRC cases). Exclusion criteria included patients who have developed any another type of cancer in the previous 5 years, those who have previously received chemotherapy or radiotherapy, subjects previously diagnosed with AA, familial adenomatous polyposis, Lynch syndrome, or inflammatory bowel disease.

Three of the 300 subjects were invalid because they did not fulfill inclusion criteria due to incorrect age or diagnosed with a non-AA. Two hundred ninety-seven subjects were finally valid and included in the study: 100 healthy individuals, 101 patients with AA, and 96 patients with CRC. We considered AA with a size of at least 10 mm or having a high grade of dysplasia or ≥20% villous component. Characteristics of the patients are shown in Table 1. CRC staging system used was the *American Joint Committee on Cancer* Tumor Nodes Metastasis classification.

**Table 1.**
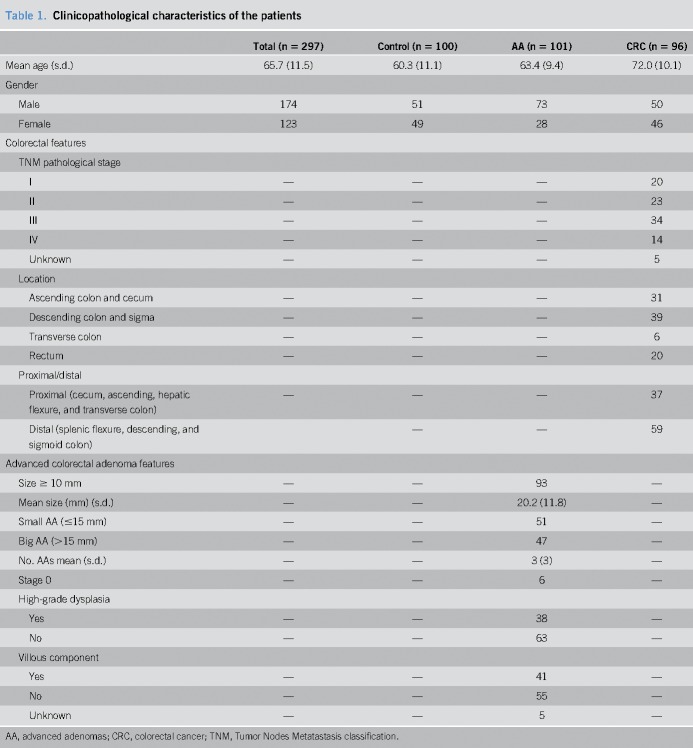
Clinicopathological characteristics of the patients

### Blood collection and plasma isolation

Peripheral blood from participants was drawn before colonoscopy and before any polyp or cancer resection, the same day of the procedure. Ten milliliters of blood were collected in a BD Vacutainer blood collection tube (Becton Dickinson, Toronto, Canada) and kept at room temperature within 12 hours of plasma isolation. Double centrifugation was required for plasma separation.

### RNA isolation

Total RNA including also miRNAs was isolated from 500 μL of plasma using mirVana PARIS kit (Ambion by Life Technologies, Carlsbad, CA) and following manufacturer's instructions. Cel-miR-39 was added as exogenous control in all samples. RNA was eluted in 30 μL of nuclease-free water and kept at −80 °C until retrotranscribed.

### miRNA expression analysis

Complementary DNA production from RNA was conducted using TaqMan MicroRNA Assays (Life Technologies). Briefly, 2 μL of RNA were retrotranscribed and preamplified in singleplex for 12 cycles with TaqMan PreAmp Master Mix (Life Technologies). The expression of each one of the target miRNAs (miR19a, miR19b, miR15b, miR29a, miR335, and miR18) (12), the housekeeping miR-1228, and the spike-in cel-miR-39 was evaluated with TaqMan MicroRNA Assays (Life Technologies) by real-time PCR in a Viia7 Real Time PCR system (Life Technologies). Both miR-1228 and cel-miR-39 were used for normalization. To calculate miRNA levels, we used cycle number at threshold (Ct) values from automatic threshold, and PCR was done in triplicate.

### Statistical analysis

#### Data cleaning (consistency and uniformity).

We used the R language (https://www.R-project.org/; version 3.4.1) and tidyverse package (version 1.1.1) for data filtering and normalization. Starting from raw data (Ct data in base 2 logarithm), samples with missing values in at least one miRNA (biomarker or normalizer) were removed. Also, samples with an outlier value in one of the miRNAs were removed. Outlier criteria take into account the different available sample groups (control or healthy, AA, CRC or both referred as advanced neoplasm); it uses boxplot.stats function from R (grDevices package). Within each group, it considered outliers if the values are beyond ±1.58 interquartile range/sqrt(n).

After filtering process, the data were normalized using a combination of exogenous and endogenous miRNAs: cel-miR-39 and miR-1228, respectively. For each sample, the formula that obtains the normalized value is −miRNAx – (cel-miR-39 + miR-1228)/2), where miRNAx is the raw value of the specific target biomarker to be normalized. Because these raw values are based to 2 logarithms, this normalization is equivalent to geometric mean of original (exponential) values.

#### Building a control vs condition classifier.

We used the caret package (version 6.0-78; https://CRAN.R-project.org/package=caret) from R to build several binary classifiers (Support Vector Machine [SVM] linear, SVM radial, and SVM polynomial) and validated them using 5-fold cross validation, repeating 10 times (13). These classifiers were trained to classify control samples against AA samples or CRC samples or both (referred as advanced neoplasm samples). Different combinations of tuning parameter values were tested for each classifier: (i) Cost (all classifiers), 5 possible values ranging from 2^−2^ to 2^2^; (ii) Sigma (radial), values ranging from 0.04 to 0.7; (iii) Scale; and (iv) Polynomial degree (only for the SVM polynomial), ranging from 10^−3^ to 10^−1^ (3 values) and 1 to 3, respectively. Furthermore, 5 different types of preprocessing were performed: (i) None; (ii) Center and Scale; (iii) BoxCox; (iv) BoxCox and Center; or (v) BoxCox, Center, and Scale. Then, for each classification objective (control vs AA, control vs CRC, or control vs advanced neoplasm), a total of 1,925 different models were generated and validated. Statistical significance was considered when *P* < 0.05 in two-side testing.

Area under the curve (AUC) was used as main performance metric to compare models (calculated with caTools package, version 1.17.1) (14). Final AUC for one model is calculated as the average over the 10 iterations of 5-fold cross validations and a cut-off of 0.5. AUC, sensitivity, and specificity values were evaluated for the best global model using the whole set. The method used for calculating confidence intervals (CIs) of AUCs was DeLong method integrated within the pROC package (15). Partial AUC when specificity and sensitivity are restricted from 0.80 to 1.00 is showed in Supplementary Table 1 (Supplemental Digital Content 1, http://links.lww.com/CTG/A3).

We used Brier score for assessing the accuracy of predictions, applicable where predictions must assign probabilities to a set of discrete outcomes. It is a measure of the mean squared error of the probability forecasts and it is expressed as the mean squared difference between a predicted probability (which must be between 0 and 1) and the actual outcome (which can take on values of only 0 and 1). The Brier score ranges from 0 for a perfect forecast to 1 for the worst possible forecast. The lower the Brier score is for a set of predictions, the better the predictions are calibrated (16).

## RESULTS

Of the 300 enrolled, 297 were successfully evaluated and 3 were withdrawn due to lack of inclusion criteria (age and diagnosed with a non-advanced adenoma). Of these 297, 100 were diagnosed by colonoscopy as healthy (controls), 101 with AA, and 96 with CRC after blood sample was taken for the study.

**Figure 1.**
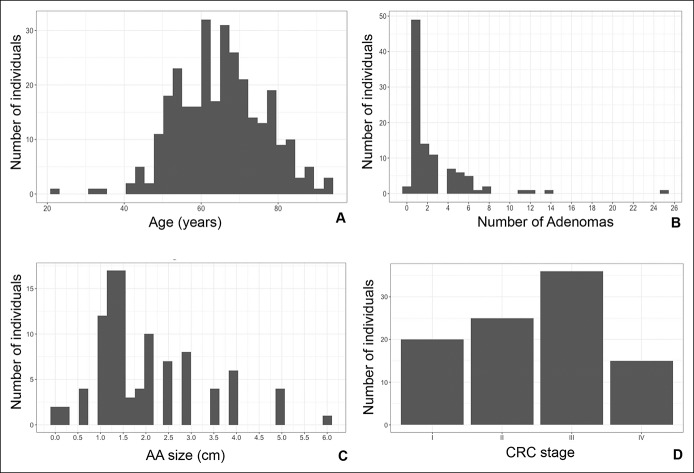
Clinicopathological features of patients. (**a**) Age distribution of the whole cohort of patients (N = 297 individuals). (**b**) Distribution of patients with AA. Number of adenomas are represented (N = 101 individuals). (**c**) Distribution of advanced adenoma size (cm) (N = 101 individuals). (**d**) Representation of patients with CRC depending on tumor stage. AA, advanced adenoma; CRC, colorectal cancer.

In more detail, we enrolled 174 (58.6%) men and 123 (41.4%) woman. Most individuals tested fell within the range of 50–80 years old and mean average was 65.7 ± 11.5 (Figure 1a).

The AA group included subjects with at least one of the following characteristics: size bigger than 10 mm as reported by pathological examination, high grade of dysplasia (HGD), and/or villous component. Mean size of AA was 20.2 mm and the mean of number of adenomas per subject was 3. The CRC group included 20, 23, 34, and 14 subjects diagnosed with stage I, II, III, and IV, respectively. In 5 cases, the stage was not determined. With respect to location, 31 cases were located in ascending colon–cecum; 39 in descending colon–sigma; 6 cases in transverse colon; and 20 cases in rectum. Number of adenomas and size (cm) were represented as bar graph to show patients’ distribution (Figures 1b,c). Distribution of CRC stages were also shown in Figure 1d.

Median age for control group (healthy individuals) was 60.3 years, 63.4 for AA, and 72.0 for CRC (Table 1). As a case–control study, CRC is more frequent in elderly population. Starting from raw data (Ct data in base 2 logarithm), 29 samples with missing values in at least one miRNA (biomarker or normalizer) were removed. Also, 47 samples with an outlier value in one of the miRNAs were removed.

**Figure 2.**
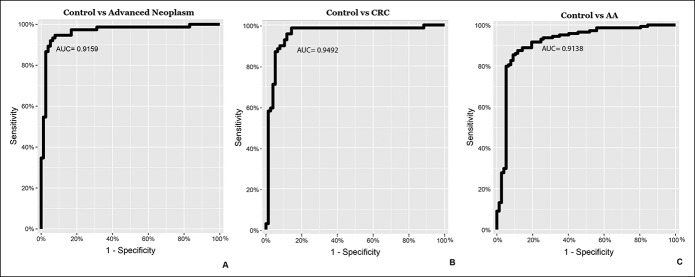
Receiver operating characteristic curve analysis for the 6 microRNA signatures. (**a**) The model control vs advanced neoplasm; (**b**) the model control vs CRC; and (**c**) the model control vs AA. AA, advanced adenoma; AUC, area under the curve, CRC, colorectal cancer.

When analyzing control vs advanced neoplasm (including patients with CRC and AA), performance of the model without cross-validation gave us an AUC = 0.92 (95% CI 0.871–0.962) with a sensitivity of 0.85 and specificity of 0.90 and positive predictive value (PPV) = 0.94 and negative predictive value (NPV) = 0.76 (Figure 2a and Table 2). According to our calculations, the cohort of patients reaches the best prediction model with an AUC of 0.79 (95% CI 0.723–0.841) after a cross-validation process, using an SVM-based model with a radial kernel and a Cost = 0.50 and Sigma = 0.44 (see Supplementary Figure 1, Supplemental Digital Content, http://links.lww.com/CTG/A3). For additional sensitivities and specificities, data are shown in Supplementary Table 2 (Supplemental Digital Content 1, http://links.lww.com/CTG/A3).

**Table 2.**
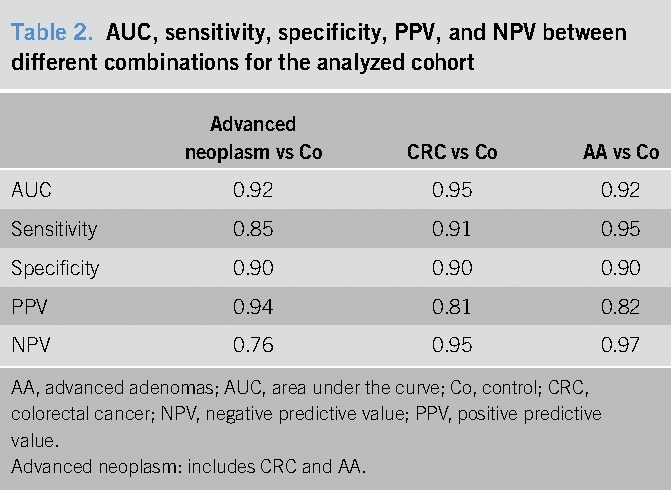
AUC, sensitivity, specificity, PPV, and NPV between different combinations for the analyzed cohort

When analyzing control vs CRC, an AUC = 0.95 (95% CI 0.903–0.991) with a sensitivity of 0.91 and specificity of 0.90, and PPV = 0.81 and NPV = 0.95 was obtained. Moreover, an AUC = 0.91 (95% CI 0.868–0.959) with a sensitivity of 0.95 and specificity of 0.90, and PPV = 0.82 and NPV = 0.97 was observed for AA vs control (Figure 2b,c and Table 2). Assuming a hypothetical screening population in which expected prevalence of CRC and AA were 1:100 and 7:100, respectively, PPV = 0.08 and NPV = 0.99 would be obtained for CRC and PPV = 0.42 and NPV = 0.99 for AA.

**Figure 3.**
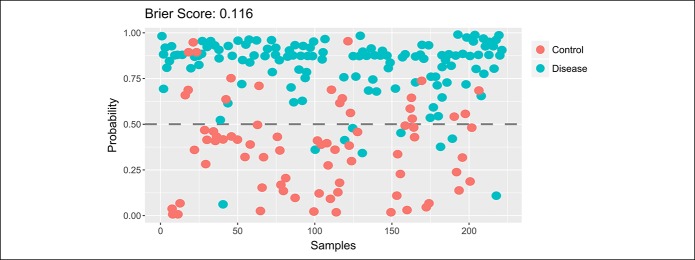
Classification by probability using Brier score measuring the accuracy of probabilistic predictions ranking from 0 (total accuracy) to 1 (wholly inaccurate). The lower the Brier score is for a set of predictions, the better the predictions are calibrated.

Figure 3 shows a model based on Brier score representing the probabilistic prediction of samples as healthy or advanced neoplasm (i.e., CRC or AA). A Brier score of 0.116 demonstrates a very good prediction forecast in separation between samples from patients (CRC and AA) and healthy.

**Figure 4.**
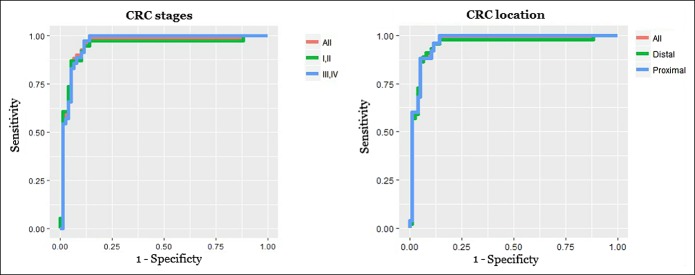
Receiver operating characteristic curve analysis for the CRC patients. (**a**) Early stages (I/II) vs Late stages (III/IV). (**b**) Proximal vs distal location. CRC, colorectal cancer.

Sensitivity did not vary significantly according to cancer stage or location within the colon. In fact, sensitivity of 6 miRNA signatures was 0.94 and specificity of 0.87 for early stages (including stages I and II), whereas it was 0.94 and 0.86, respectively, for late stages (III and IV). Likewise, sensitivity/specificity was 0.88/0.94 for proximal CRC and 0.89/0.94 for distal CRC (Figure 4 and Table 3).

**Table 3.**
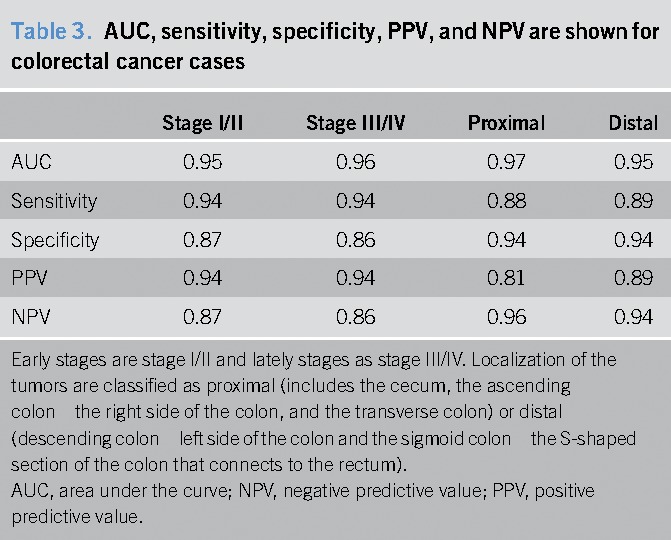
AUC, sensitivity, specificity, PPV, and NPV are shown for colorectal cancer cases

Individual evaluation of miRNAs analyzed, miRNA15b and miRNA29a are consistently showing its high significance when contributing to distinguish control vs advanced neoplasm group. In this cohort of patients, miRNA15b, miRNA29a, and miRNA335 analyzed independently showed statistically significant *P* values as obtained by means *t* test (7.9e-5, 5.7e-5, and 0.013, respectively). This was observed in univariate, logistic regression models and also SVM models.

## DISCUSSION

CRC is an ideal target for population screening because it is a prevalent disease in which detection and treatment at an asymptomatic stage lead to a mortality reduction as it has been demonstrated in different screening strategies.

Several randomized controlled trials demonstrated that screening with guaiac FOBT and flexible sigmoidoscopy are effective in reducing CRC mortality by approximately 16%–25% (17) and 26%–38 % (18–20), respectively, and even stronger reductions in incidence and mortality are expected for FIT and colonoscopy screening due to higher diagnostic accuracy.

Consequently, with time, big health care organizations have been changing recommendations. While in 2008, US Preventive Services Task Force included only 3 strategies: FOBT, flexible sigmoidoscopy, and colonoscopy, and in 2016, the number of recommendations was updated to 7 also including FIT-DNA. The largest study assessing sensitivity and specificity of FIT-DNA test called Cologuard from Exact Sciences (Madison, WI) demonstrated a sensitivity of 92% for CRC and 42% for AA at a specificity of 87% using colonoscopy as gold standard (21). Moreover, in 2016, the Food and Drug Administration approved a blood test to detect circulating methylated SEPT9 DNA (https://www.accessdata.fda.gov/cdrh_docs/pdf13/p130001c.pdf) with a low sensitivity (48%) for detecting CRC (22). FIT characteristics vary between different studies although meta-analysis estimates an overall cancer sensitivity and specificity of 79% and 94% but FIT lacks the capacity to detect AA (23). Recent publications comparing diagnostic performance between various quantitative FITs concluded that small differences in results can be overcome by appropriate threshold adjustments to yield a desired level of specificity (24,25).

Early detection of AA remains an unmet need as there is no marketed test available with promising sensitivity for these lesions becoming cancerous.

The development of more sensitive and specific tests to detect CRC and AA is a reality. The need to increase the adenoma detection rate for CRC screening is patented in devices that mechanically or optically try to improve conventional colonoscopy. Blood-based test may offer advantages compared to colonoscopy, because it does not require intensive time commitment (bowel preparation, procedure itself, and recovery), and FIT, as the person should not need to handle their feces at home with logistic difficulties. It is well known that minimally invasive tests will achieve higher adherence over time taking into consideration availability, costs, and patient–clinicians preferences (26,27).

In this study, we validated a signature of 6 miRNAs as biomarkers for early detection of CRC in plasma samples from a cohort of patients with CRC and AA, and healthy individuals. Our results demonstrated a high sensitivity and specificity using real-time PCR technology, an easy technique to be performed in hospitals and diagnostic laboratories. The signature includes miRNAs previously described and related to CRC development by other authors (12). Improving sensitivity and specificity is highly desirable for efficient population-based screening with good adherence rates. Very promisingly, the signature is able to detect AA with high sensitivity and specificity.

Herein, a recent publication gave insights about miRNA19a promoting CRC development when bound directly to the 3′-untranslated region of TIA1 mRNA promoting cell proliferation and migration of CRC cells (28). Huang et al. (29) also showed that miRNA19a plays an important role in mediating epithelial to mesenchymal transition and metastatic behavior in CRC, serving as a potential marker of lymph node metastasis. Similarly, miRNA19b-3p promotes colon cancer proliferation and chemoresistance to oxaliplatin by targeting SMAD4 (30). On the other hand, experiments from some authors demonstrated that transcriptional repression of miRNA15b-5p by SIRT1 could suppress CRC metastasis showing a roll of this miRNA as potential target for therapy (31). Very importantly, Tang et al. concluded that an increased expression of miRNA29a targets KLF4, which highlights the potential of miRNA29a inhibitors as novel agents against CRC metastasis (32). In fact, data obtained in our cohort of 297 individuals concluded that miRNA15b and miRNA29a are significantly contributing to the predictive model through an overexpression in patients with AA or CRC compared to those who are healthy.

The proposed miRNA signature here presented is based on the combination of several of these miRNAs, some of them previously associated to colon cancer diagnosis (12), prognosis, or prediction to response to therapy. Molecular mechanisms clarifying how these miRNAs are acting as oncomiRNA must be elucidated.

With the proposed model integrating values from these specific miRNAs, the signature possesses a sensitivity of 91% and specificity of 90% for CRC and sensitivity of 95% and specificity 90% for AA. According to these values, we present a high-performance blood-based test. Main advantages of this test are a minimally invasive test for CRC screening and outstanding sensitivity and specificity for AA.

Sensitivity and specificity in detecting proximal and distal advanced neoplasia are similar for our miRNA signature. In view of the findings of the current results, this miRNA signature faces the raising burden of proximal colon cancer. Very importantly, detection rate for CRC in early stages (I and II) is comparable with late (III and IV). Both characteristics offer a superiority to FIT.

Although some authors demonstrated a better diagnostic performance for the relative detection of CRC in the distal colon than that in the proximal colon when using fecal test (33), other showed that FIT is equally sensitive for proximal and distal neoplasia (34). Niedermaier et al. (23) conducted a systematic review and meta-analysis of 10 studies (23,34–41) performed in screening population with the aim to evaluate performance of FIT depending on stratified by left or right side where advanced neoplasia was located. This meta-analysis estimated a FIT sensitivity for CRC and AA detection of 63% and 22% in the right colon and 67% and 32% in the left colon and rectum, concluding that there are small differences in CRC detection and pronounced differences in AA detection between left- and right-sided neoplasia.

In the work we presented in this manuscript, using a miRNA signature, we demonstrated equally effectiveness to detect lesions in both locations proximal and distal.

We consider that high sensitivity is the most important attribute for cancer screening test but high specificity is also important, as otherwise it could affect participants obtaining false positive results when in fact they are healthy, due to low prevalence of CRC, which ultimately will increase anxiety in patients while increasing costs in unnecessary colonoscopies. The percentage of US residents up-to-date on CRC screening has not increased appreciably since 2010 and remains at approximately 62.4% in 2015 (42). The National Colorectal Cancer Roundtable has established a goal of 80% adherence on CRC screening by the year 2018.

Very interestingly, this miRNA signature is able to detect cancers occurring at the right side, which is an advantage because incidence of right-sided colon cancers has been progressively increasing over recent decades (43). Colonoscopy is more effective in preventing left-sided as compared with right-sided and blood-based tests could represent a solution in early detection for a demonstrated poorer prognosis and worse response to targeted therapy of right-sided cancers (44).

The COLONPREV study, a randomized, controlled trial, demonstrated that patients are more likely to participate in screening programs using a noninvasive test as FIT compared to those choosing colonoscopy (45). Importantly, on screening examination, the number of subjects in whom CRC was detected was similar by the 2 methodologies although more adenomas were identified in the colonoscopy group. The result of the COLONPREV trial suggested that innovative noninvasive or minimally invasive methods are required for AA detection. Our miRNA signature evidenced a robust method for AA detection in plasma samples.

Here we presented a validated model in which a miRNA signature could be integrated in a minimally invasive method for CRC screening purposes after further prospective clinical validation. One of the main factors for success in screening programs and reducing CRC mortality is to achieve a high participation. Further studies in a real CRC screening population are required to validate the model presented in this article. Additionally, a comparison or even combination with FIT screening method will be of great value. Moreover, prospective studies will help to gain evidence about appropriate follow-up of positiveness of blood-based test although diagnostic colonoscopy is negative.

## CONFLICTS OF INTEREST

**Gurarantor of the article:** Rocio Arroyo, BSc.

**Potential competing interests:** M.H.‐V. and S.D‐S. contributed equally to this work. M.S. and R.A. are co-senior authors. M.H.-V., A.C.M., R.P.-P., and R.A. are Amadix employees. Rest of the authors declare no conflict of interest.

**Specific author contributions:** M.H.-V., A.C.M., R.P.-P., M.G., and R.A. designed, wrote, and edited the manuscript. S.D.-S., E.V.-N., and M.M. performed molecular analysis. M.D.-C., J.C., M.S.D., L.B., A.L., R.J., V.H., E.Q., and A.C. participated in sample collection and study design. J.L. and I.M.-A. performed statistical analysis. All authors made comments and approved the manuscript.

**Financial support:** The present work was funded by Advanced Marker Discovery, S.L. (Amadix) and its private resources. CIBEREHD is funded by the Instituto de Salud Carlos III. Dra. Gironella's research group also acknowledge the support of grant from Instituto de Salud Carlos III (PI17/01003, co-funded by FEDER-European Union) and of CERCA Programme (work developed in part at Centro Esther Koplowitz, Barcelona).

**Study Highlights**

**WHAT IS KNOWN**

✓ miRNA signatures in biological fluids can facilitate earlier detection of the tumors.
✓ miRNA could be diagnostic biomarkers in CRC but validation studies are required.

**WHAT IS NEW HERE**

✓ miRNA could be useful as diagnostic biomarkers.
✓ A signature of specific miRNAs is able to distinguish healthy subjects from those with CRC and AA.
✓ A signature of 6 miRNAs could be used for CRC screening purposes.

**TRANSLATIONAL IMPACT**

✓ Reducing cancer incidence and mortality.
✓ Detecting CRC even precursor lesions earlier.
✓ Blood-based and minimally invasive test.
✓ Achieving higher participation and compliance in CRC screening programs.

## Supplementary Material

SUPPLEMENTARY MATERIAL
