# Supplementary figures and images for: Plasma MicroRNA Signature Validation for Early Detection of Colorectal Cancer
Source: Clin Transl Gastroenterol. 2019 Jan 25;10(1):e00003. doi: 10.14309/ctg.0000000000000003 (PMC6369870; doi:10.14309/ctg.0000000000000003)

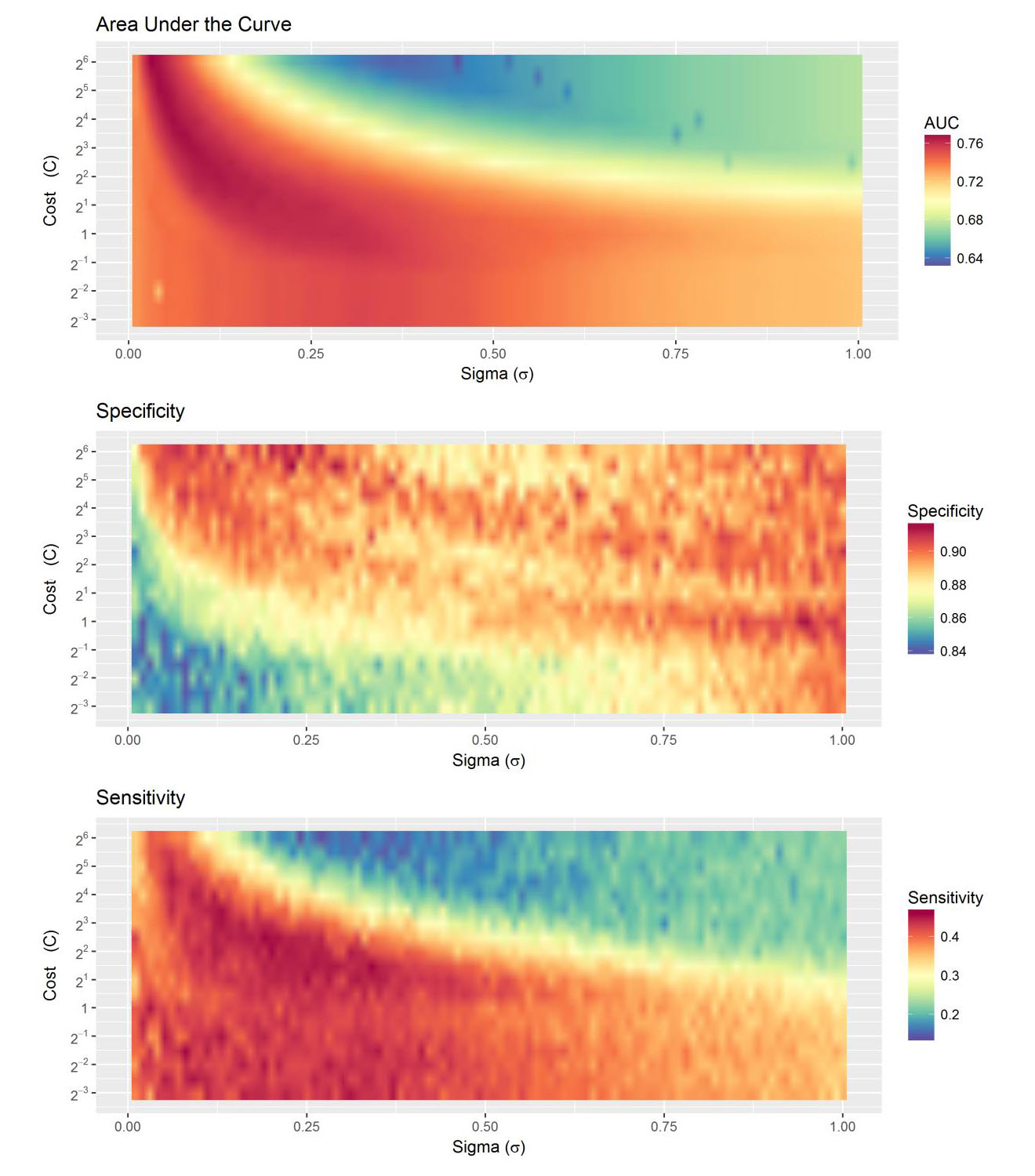

Supplement: SUPPLEMENTARY MATERIAL [file ct9-10-e00003-s001.jpg]
